# Is This All COVID-19′s Fault? A Study on Trainees in One of the Most Affected Italian Cities

**DOI:** 10.3390/ijerph192013136

**Published:** 2022-10-12

**Authors:** Paola Manfredi

**Affiliations:** Department of Clinical and Experimental Sciences, University of Brescia, 25123 Brescia, Italy; paola.manfredi@unibs.it

**Keywords:** mental health, coping strategies, well-being, burnout, COVID-19, anaesthesia–resuscitation residents, psychiatry residents

## Abstract

Many studies have investigated the state of the health of healthcare workers during the acute period of the pandemic. Yet, few studies have assessed the health of such professionals after the pandemic and in a less dramatic period. This study involved a particular sample represented by residents in anaesthesia–resuscitation and psychiatry at a university in northern Italy particularly affected by the pandemic. The objectives were to investigate some indicators of health and well-being and compare the two groups of trainees. Using Google Forms, the following tests were proposed: the General Health Questionnaire, Maslach Burnout Inventory, Subjective Happiness Scale, Satisfaction with Life Scale, Coping Inventory for Stressful Situations, Brief Resilience Scale, State-Trait Anxiety Inventory, as well as an ad hoc questionnaire. A qualifying element of the work was the discussion of the results with the trainees. Various strengths have emerged, such as high values of resilience and job satisfaction; a positive assessment of the support received from the work team; an articulate use of coping strategies; and good levels of happiness and satisfaction with life, in both specialities. However, a widespread anxiety also emerged, which appears to be more attributable to concerns about professional evaluation, rather than the pandemic itself. In summary, the trainees seem to have found a fair amount of personal balance, whereas the relationship with the patient seems to be more compromised. In the comparison between specialities, the only significant differences are the levels of depersonalisation and resilience, both of which are higher in anaesthetists.

## 1. Introduction

During the pandemic, many studies have assessed the well-being vs. the discomfort of workers, particularly healthcare workers. Various studies, in different parts of the world, have highlighted the presence of anxiety, depression, burnout, physical symptoms and psychological distress among healthcare workers. For example, psychological distress appears to be quite significant to 78% of Japanese health workers [1]. In Australia and Denmark, serious depression was experienced by 13.5% of the respondents, anxiety was present in 12.9% and stress in 13.4% [2]. In another Australian study, psychological distress (anxiety, depression and stress) is found in a quarter of the respondents [3]. In India and Singapore, a multicentre study has revealed moderate to very severe depression in 5.3% of the sample, moderate to very severe anxiety in 2.2% and stress in 3.8%. Percentages reflecting the presence of physical symptoms are even higher: headaches were present in 32.3% of the subjects, and 33.4% of them lamented more than four physical symptoms [4]. In a study in Oman, almost one third of healthcare workers reported moderate to severe anxiety and a low level of well-being [5]. In a New York-based study, 30% of healthcare workers in the pooled analysis had experienced anxiety [6].

Medium–high levels of emotional exhaustion (28–38.3%), displayed high depersonalisation (25–31.05%) and low to medium levels of personal accomplishment (42–46.5%) [7,8,9] were found among Italian healthcare workers. Furthermore, among these subjects, the presence of physical symptoms and somatisation was encountered in a large percentage, between 45% [8] and 71% [10]. Additionally, depressive symptoms—from 9% [9], to 24.73% [10] to 26.6% [11]—anxiety—from 19.8% [9], to 21.5% [10] 50.1% [11]—and stress—from 21.9% [9], to 33.4% [10] to 55% [12]—were noticed.

What has been less studied is the trend over time, i.e., whether these patterns have changed and in which direction. We have experienced the pandemic through several waves, and the devastating impact of the early days has been followed by a less dramatic period, both due to a reduction in infection and a greater knowledge of the disease, the development of vaccines and more effective treatments [13,14,15,16,17,18,19]. On the other hand, new variants have taken over [20], some restrictions have been reintroduced and there is a growing feeling that we will have to live with this virus rather than hope to eradicate it. The data in the literature differs, depending on the period and population considered. A study carried out in 2021 showed that Americans, after receiving the first dose of the vaccine, showed reduced levels of mental distress, on both severe and mild depression [21]. Another study comparing the first and second pandemic waves, among the Italian population, confirmed the decrease in anxiety [22]. In a three-wave longitudinal study with a sample of Italian adults, it was found that psychopathological symptoms and psychological well-being co-varied with the intensity of the COVID-19 pandemic and the associated social restrictions [23]. In a study from the same period, i.e., 2022, high levels of stress, anxiety and depression were observed in a sample of Mexican students [24].

Our goal consists of checking the current state of health vs. disease in a particularly vulnerable sample, i.e., young doctors in training, in an area greatly affected by the pandemic. The interest is related not only to the fact that the literature has shown that young age exposes a greater extent people to the negative consequences of the pandemic [8,10], but also to the specific role of young professionals in the protection of public health. It is difficult for a doctor to take care for patients properly when he/she is not in good health, both physically and psychologically.

In this study, we investigated some indicators of health vs. suffering in trainees in anaesthesia–resuscitation and psychiatry residents. They shared the same age and belonged to a geographical area in north-west Italy, which was particularly affected in the first wave, but they experienced a more or less protected situation, with respect to any contact with patients. In fact, while the former workers were almost always on the front line, with a greater time commitment than usual, the latter workers were mainly in protected situations and with a reduced load, since fewer patients were admitted to their ward. A qualifying element is that, as the study was part of a training course, it was possible to use various tools to get a more articulated picture, but above all, it was possible to share and reflect with the trainees themselves about a goal to check for possible correlations between the indicated variables. In particular, our hypothesis was that the state of the well-being and health of the trainees was correlated with greater resilience abilities, task-oriented coping, professional gratification and was inversely correlated with depersonalisation, emotional exhaustion and emotional coping. Furthermore, it was hypothesised that depersonalisation and emotional exhaustion (burnout) were positively correlated with avoidance-oriented and emotion-oriented coping styles and negatively with task-oriented style. Finally, it was assessed whether the variables considered were influenced by belonging to different specialities.

## 2. Materials and Methods

### 2.1. Procedure

The research was part of a training course addressed to 2nd, 3rd and 4th year trainees in anaesthesia–resuscitation, and separately trainees in psychiatry. After the most critical period of the pandemic, the aim was to assess the state of the well-being, or the lack of it, of the residents. The residents were involved in this project using the administration of tests and the sharing of the results in aggregate form as the chosen modality. The heads of the respective specialities authorised the work, while it was not necessary to ask for consent from the ethics committee as the compilation was anonymous. The questionnaires were prepared to be filled in on Google Forms, anonymously. The residents were asked to indicate a code, consisting of the day and month of the birth of their mother and father, in order to preserve anonymity and potentially make it possible to compare data in the follow-up. Before completion, informed consent was requested. The processed data were then presented to each group of residents separately. The work took place between November 2021 and March 2022.

### 2.2. Sample

The sample consisted of anaesthesia–resuscitation and psychiatry residents at the University of Brescia. The inclusion criterion was: their enrolment in the 2nd, 3rd or 4th year of the specialities of anaesthesia–resuscitation or psychiatry at the University of Brescia. The exclusion criterion was: having stayed abroad in the previous two years for more than 3 months. The number of subjects meeting the requirements was 96; the number of subjects who filled in the questionnaires was 90. The sample consisted of 63 trainees enrolled in the speciality of anaesthesia–resuscitation and 27 in psychiatry. Six anaesthesia–resuscitation residents did not fill out the protocols, while all psychiatry residents did. The age range was from 26 to 39, with the mean being 29.50 and the SD was 2.523. Females made up 43.3% of respondents and males made up 56.7%.

### 2.3. Instruments

The set was composed of several tests. Moreover, an ad hoc questionnaire was prepared (attachment 1, Supplementary Materials); in particular, specific behaviours and experiences during the pandemic were investigated. For example, the items of the questionnaire were: “I feel that my psychic well-being has deteriorated” and “Did you generally feel qualified for the job you were asked to do?”

#### 2.3.1. General Health Questionnaire

The GHQ 12 is a questionnaire that provides a global measure of the psychopathological state by differentiating a subjective state of well-being and a psychological distress level. For example, a question of the questionnaire was: “Have you recently been losing confidence in yourself?”. The best cut-off point for the GHQ 12 was ≥14 [25]. The scores were divided into low and high. The validated Italian version of the GHQ 12 [26] was used. In the literature, the GHQ 12 presents a satisfactory internal consistency; in our sample, Cronbach’s α was low (0.53).

#### 2.3.2. Maslach Burnout Inventory

Burnout was detected with the Italian version of the Maslach Burnout Inventory developed by Sirigatti and Stefanile [27]. The MBI questionnaire consists of 22 items. Each item is rated on a 7-point Likert scale (0 = ‘never’ to 6 = ‘every day’). The questionnaire consists of three subscales: emotional exhaustion (9 items), depersonalisation (5 items) and personal accomplishment (8 items). For example, an item of the emotional exhaustion subscale was “I feel emotionally exhausted by my work”; the item “I don’t really care about what happens to some users” belongs to the depersonalisation subscale; and an example of an item from the personal accomplishment subscale is “I feel full of energy”. According to Sirigatti and Stefanile’s categorisation of scores, the emotional exhaustion subscale scores are divided into low (≤14), medium (15–23) and high (≥24); the depersonalisation subscale scores are divided into low (≤3), medium (4–8) and high (≥9); and the personal accomplishment subscale scores are divided into low (≥37), medium (30–36) and high (≤29). In our sample, Cronbach’s α was 0.71, thus confirming a satisfactory internal consistency.

#### 2.3.3. Subjective Happiness Scale

The Subjective Happiness Scale (SHS) [28] has been used to measure the overall level of perceived happiness. The SHS is a self-assessment measure consisting of four items on a 7-point scale (from 1 = “I’m not a very happy person” to 7 = “I’m a very happy person”). Two items evaluate the general perception of happiness, e.g., “I generally consider myself a not very happy/very happy person”, and the other two evaluate the perception of happiness compared to others, e.g., “Compared to most of my peers, I consider myself to be: less happy/happier”. The total score is obtained by adding the scores of the individual items, reaching an overall score that can range from 4 to 28. Higher scores represent a greater overall happiness. The Italian version was used [29]. In our sample, Cronbach’s α was 0.79.

#### 2.3.4. Satisfaction with Life Scale

The Satisfaction with Life Scale (SWLS) [30] is a self-assessment scale that measures general life satisfaction. The subjects are asked to complete the questionnaire by filling in a 7-point Likert scale (1 = strongly disagree to 7 = completely agree). An example of an item on the scale is: “If I could live my life all over again, I would change almost nothing”. The total scores on the scale are calculated by averaging the responses to the five items, and they therefore range from 1 to 7. A higher score indicates a greater overall satisfaction with life. The Italian version of the scale, which was used for the test, was edited by Di Fabio and Busoni [31]. The results of the factorial structure confirm, also for the Italian version, a one-dimensional scale structure [32]. In our sample, Cronbach’s α was 0.86.

#### 2.3.5. Coping Inventory for Stressful Situations

The Coping Inventory for Stressful Situations (CISS) is a self-describing coping assessment instrument based on Endler’s interactive model of stress, anxiety and coping [33]. It is a multidimensional coping tool consisting of 48 items divided into three scales: T = task-oriented, E = emotion-oriented and A = avoidance-oriented. The avoidance scale comprises two subscales: D = distraction and SD = social diversion. For each item, the subject is asked to indicate the frequency on a 5-point scale, ranging from 1 ‘not at all’ to 5 ‘very much’. The Italian version of Sirigatti was used [34]. The results were converted into T-scores according to the Italian tables. Following the handbook, in the Italian adaptation [34], the T-scores were divided into these ranges: extremely above average, very above average, above average, slightly above average, average, slightly below average, below average, very below average and extremely below average. In our sample, Cronbach’s α was 0.85

#### 2.3.6. Brief Resilience Scale

The Brief Resilience Scale [35] assesses a unitary construct of resilience, intended as the perceived ability to bounce back or recover from stress. The scale consists of 6 items and is scored on a 5-point Likert scale. For example, an item on the scale is: “I tend to bounce back quickly after hard times”. The possible score range is from 1 (a low resilience) to 5 (resilience). A low and high resilience are defined as a BRS score of 4.3, respectively [36]. The Italian translation of the Brief Resilience Scale was used [37]. The measure of internal consistency in our sample was good, with Cronbach’s α = 0.86.

#### 2.3.7. State-Trait Anxiety Inventory

The State-Trait Anxiety Inventory (STAI) [38,39,40] is a reliable and valid research instrument which detects the intensity of feelings of anxiety; it distinguishes between state anxiety, a temporary condition experienced in specific situations, and trait anxiety, a general tendency to perceive situations as threatening. Individuals are asked to specify, on a 4-point Likert scale from ‘not at all’ to ‘very much’, to which extent they usually perceive each of the 20 indicated feelings. The range of scores for each subtest is 20–80, with higher scores indicating a greater level of anxiety. As indicated in other studies, the cut-off at a score of 40 was adopted for both scales [41,42]. This value corresponded to the point at which false positive and negative results were minimal. The measure of internal consistency was good, with Cronbach’s α = 0.82 for the STAI I and Cronbach’s α = 0.89 for the STAI II.

### 2.4. Statistical Analysis

The data analysis was performed using SPSS 26 statistics software. Kolmogorov–Smirnov was applied to each test, and since the distribution was not normal and the scales are not interval or ratio scales, non-parametric statistics, such as the Kruskal–Wallis test, the Mann–Whitney test, the Chi-Square test, Wilcoxon’s rank test and correlations among the variables by the Spearman’s rank, were used. In the presence of a significant Kruskal-Wallis test result (*p* < 0.05), a post-hoc analysis was performed to examine the specific groups of interest, taken in pairs (Dunn’s test). The data were described as frequencies or percentages, expressing the median and the minimum and maximum values. However, as the entire sample is quite copious and to facilitate comparisons, the mean and standard deviation were also calculated. A two-sided α level of 0.05 was used for all tests.

## 3. Results

### 3.1. Descriptive Statistics

The descriptive analyses have been summarised in the tables below (Table 1, Table 2 and Table 3). Table 1 shows the percentages of the items of the ad hoc questionnaire.

The following table (Table 2) shows the median, percentiles, minimum and maximum values and the mean and standard deviation for the proposed tests.

The following table (Table 3) shows the percentages compared to the cut-off points, differentiated for the three samples.

### 3.2. Correlations

The STAI I and STAI II correlated with each other and correlated positively (*p* = 0.01) with emotional exhaustion, depersonalisation and emotion-oriented coping, and negatively with task-oriented coping. The STAI I also correlated (*p* = 0.05) with personal accomplishment. Table 4 and Table 5 show the other significant correlations.

### 3.3. Differences

Comparing the samples by speciality, only two statistically significant differences emerged, relating to resilience (*p* = 0.010) and depersonalisation (*p* = 0.007) values. Table 6 shows the mean and standard deviation values.

When differentiating the sample of the trainees according to gender, differences emerged in the score distribution on State I (*p* = 0.040) and State II (*p* = 0.016). Considering the sample’s psychological well-being separately, differences emerged with regard to State II (*p* = 0.044) and personal accomplishment (*p* = 0.013): females showed more anxiety and less personal gratification. In the group of psychiatrists, the sex variable did not discriminate the sample. When comparing State I and State II scores, a significant difference (*p* = –0.009) emerged in the anaesthetists’ group, where trait anxiety was lower than state anxiety, whereas no difference was registered in the psychiatrists’ sample. The experience of not being able to offer the most appropriate care to everybody due to the pandemic characterised the entire sample, with respect to emotional exhaustion *p* = 0.05, depersonalisation *p* = 0.01 and distraction *p* = 0.047; such values increased progressively from subjects who had such an experience (group 1), subjects who had an occasional experience (a few times—group 3) or subjects who had no experience (group 2). The experience of not being able to offer the most appropriate care to everybody was lived more by anaesthetists than by psychiatrists (*p* = 0.005); considering only anaesthetists from the pairwise comparisons, significant differences emerged for emotional exhaustion between groups 2 and 1, with *p* = 0.048, and between groups 3 and 1, with *p* = 0.036; with respect to depersonalisation, there was a significant (*p* = 0.012) difference between groups 2 and 1; and finally with respect to the evaluation of psychological well-being, there was a difference (*p* = 0.031) between group 3 and 1.

When comparing the sample of anaesthetists with respect to the experience of communicating a patient’s worsening health condition to family members, some significant differences emerged with respect to the distribution of avoidance (*p* = 0.000), social diversion (*p* = 0.005) and distraction (*p* = 0.001). In particular, there were significant differences with respect to distraction between group 1 (those who reported worsening health conditions) and group 2 (those who never reported worsening health conditions) with *p* = 0.001; group 2 with respect to group 3 (those who sometimes reported worsening health conditions) with *p* = 0.020; group 2 and group 3 differed with respect to social diversion with *p* = 0.006; group 2 and group 3 differed with respect to avoidance with *p =* 0.003; and groups 2 and 1 with *p* = 0.001. Evaluating the sample of the anaesthetists with respect to the assessment of their own professional competence, significant differences emerged with respect to the SHS (*p* = 0.003), GHQ 12 (*p* = 0.041), STAI I (*p* = 0.028) and STAI II (*p* = 0.002). The post-hoc analysis revealed a difference between the group of those who rated their professional competence as adequate enough and those who did not think they were sufficiently prepared, with the following adjusted significance: the SHS (*p* = 0.002), GHQ 12 (*p* = 0.036), STAI I (*p* = 0.027) and STAI II (*p* = 0.002). Table 7 shows the values of the mean standard deviation, median, minimum and maximum.

### 3.4. Discussion of Results with Trainees

As agreed, the results of the tests, separated according to each speciality, were communicated to the trainees in group meetings. Some of their reactions are reported below. When reporting to the anesthetists, they were surprised by such high rates of anxiety, and, quite surprisingly, that the pandemic situation was not the cause of their anxiety, at least in their verbalizations. Their representation of reality was clear, in fact some of them noted that from one shift day to another, they could find that ‘half a ward changed’ due to the high number of deaths. The occurrence of patient deaths was, so to speak, already foreseen when choosing the speciality in anaesthesia–resuscitation, and the high number of deaths did not seem to be a destabilizing factor: “if we were not able to cope with the death of patients, we could not do this job”. In addition, the feeling that, in such a situation, they were united by what was happening everywhere in the world seemed to be a normalising element: “it’s like this everywhere, not just for us”. The fact that anaesthesia trainees experienced a high number of deaths in their own hospital, whereas psychiatry trainees were informed about mortality through the media, did not seem to be a differentiating factor in the sample.

When investigating the possible sources of anxiety, they were unanimously traced back to personal and work-related reasons, on which the pandemic had only a tangential influence. From a didactic point of view, the pandemic offered opportunities for intense learning, giving trainees the chance to extensively experiment techniques and practices that are more restricted in an ordinary course. On the other hand, many of the subjects faced clinical situations without having acquired or consolidated their skills yet. The fear (anxiety) of not being up to the situation and, in particular, of not being positively evaluated by their tutor, was significant, especially as they frequently changed tutors, thus they were continually exposed to new evaluations. Another element that emerged from the group to downplay the significance of the elevated levels of anxiety returned by the questionnaires was that anxiety was not perceived as detracting from their performance, but was compatible with the normal management of their commitments.

With regard to the relationship with the patient in particular, it should be noted that for anaesthesia–resuscitation residents, the data were largely unexpected. Indicative was the speech that underlined how, in comparison with other medical specialities in which the patient could be an active part in the relationship, favouring the attention of the doctor to the patient, for anaesthetists the responsibility for the relationship relies entirely on the doctor and does not involve the patient, since the latter is predominantly sedated. Avoiding all too easy ironies, we believe that this can be read as a different way of understanding the relationship, in which physical care, attention to the patient’s well-being and respect for his bodily integrity are assumed as the parameters of a good relationship. Although this may seem very far from, or limited with respect to, the idea of the doctor–patient relationship as commonly understood, in their eyes it is plausible. The emergence of the trainees having a ‘good faith’ produces some astonishment and a certain regret for the data on depersonalisation and emotional exhaustion.

The group of psychiatrists, on the other hand, expected the detected levels of anxiety, and some stressed that they would prefer to be more serene, but even in this group the pandemic was not a significant cause of anxiety. During the pandemic, there was less work than usual: almost all residents remained in hospital wards and they delayed the admission of non-critical patients, supporting them with telephone consultations. The severity of the patients admitted to psychiatry did not put the residents in a position to process the anguish of death or any significant bereavement with them. According to the residents, the patients tended to be “closed in on themselves and scarcely affected by the pandemic”. Only in a few patients did the pandemic modulate somatic delirium themes and, in a few of them, a persecutory reading of the pandemic emerged. What the patients suffered most from was home isolation, especially in the presence of family conflicts. During lockdown periods, the certification of psychiatric pathology allowed patients to leave, thus alleviating the discomfort of isolation. Moreover, for what concerns psychiatrists, reasons for disquiet were ascribed to bureaucratic tasks and delays in the provision of counselling visits: “I like working with patients; if you have a good interview, if you get meaningful answers, you can be satisfied. It’s the bureaucracy, the confrontation with colleagues and relatives that is very heavy”. Another example: “The work with patients is only one part, but the ‘corollary’ takes a lot of time and is heavy”. Reasons for stress were ascribed to relations with superiors and colleagues, especially those who feared contagion and wanted to avoid counselling. On the other hand, they themselves shared concerns about the health of staff members who might be in contact with infected patients. With respect to the burnout variables—depersonalisation and emotional exhaustion—there was less surprise, but also less regret.

## 4. Discussion

The first distinctive feature of the sample is the presence of elements of a different polarity. There are several strengths, such as high values of resilience and job satisfaction; a positive evaluation of the support received from the work team; an articulate use of coping strategies; and good levels of happiness and satisfaction with life, in both specialities. Still, among the positive notes, it should be noted that about one third of the subjects were able to take the painful experience of the pandemic as an opportunity to appreciate life more and build deeper relationships. On the other hand, a similar percentage of the sample felt they were living more in the day-to-day and registered a diminished desire to go out. Given the young age of the sample, it seems to us that both of these findings carry some weight and may signal that what was experienced in the pandemic has left a mark on these individuals. With regard to quality of life and mental well-being (GHQ 12), the absence of problems concerns a limited group of residents and anxiety also seems to affect a large percentage of both specialities. The assessment of anxiety in studies on pandemics has generally been carried out with questionnaires that are more streamlined than the STAI, so, while emphasising that the comparison is not entirely correct, we note that in the literature the percentages are lower, while in a study [22] with the same instrument, the presence of anxiety in our sample is in line with what was found in the second administration of the study aforementioned. As for the coping strategies, it should be noted that our sample makes use of a variety of strategies. What has already been found in the literature on the relevance of social support [43,44,45] is confirmed, while the use of religious coping mechanisms, which instead emerged in other research [46], is not confirmed by our study. Instead, the use of music as a means for coping with highly emotional situations during the pandemic is noted (and this is an original finding, not investigated in similar studies). Experiencing burnout is widespread among medical professionals and has been estimated at between 14.7% and 90.4% in recent years [47]. Our sample is no exception, but it presents some peculiarities: compared to other Italian studies (7, 9), emotional exhaustion is in line with the findings in the literature, while depersonalisation and personal accomplishment appear much higher.

With respect to the hypotheses that the state of the well-being and health of the residents correlates with a greater resilience, task-oriented coping and personal accomplishment, these are partly confirmed. The tests with the highest correlations are the GHQ 12 and SHS. In addition to being correlated with each other, they also correlate with the subjective assessment of the deterioration of physical health and well-being, the assessment of subjective satisfaction, state anxiety and trait anxiety, resilience, a task-oriented coping style and an emotion-oriented coping style. It is also interesting to point out that both the GHQ 12 and SHS correlate with avoidance-oriented coping and social distraction, but the former correlates inversely while the latter correlates positively. The hypothesis of correlation of the GHQ 12 with emotional exhaustion is also confirmed, but not with depersonalisation nor with personal accomplishment, whereas the SHS also correlates with the latter. Even the simple subjective perception of the worsening of well-being conditions correlates with the indicators considered above, except for personal accomplishment and the different coping styles, with the exception of emotion-oriented coping. The only element without any correlation is distraction. Partially confirmed is also the hypothesis on burnout and coping styles. Emotional exhaustion and depersonalisation positively correlate with an emotion-oriented coping style and negatively with a task-oriented coping style with a personal accomplishment, but there are no correlations with avoidance-oriented coping, distraction and social diversion. Furthermore, as one might logically expect, the presence of anxiety is positively correlated with the use of emotion-oriented coping and negatively with task-oriented coping. Typically, emotion-oriented and avoidance-oriented coping styles are considered less effective and have been associated with increased levels of emotional exhaustion, whereas low scores on the depersonalisation subscale and high scores on the MBI personal accomplishment subscale have been strongly associated with task-oriented coping styles [48]. On the other hand, two Italian studies report a reduction in psychological distress related to the use of negative coping styles, such as blocking emotions and unpleasant thoughts [49,50]. Our sample thus seems to support these latter findings, at least as far as avoidance strategies.

With regard to the comparison between specialities, some interesting data emerged. Contrary to the data in the literature [8,51] and to what seems logical to expect, the comparison between those who were directly on the front line, such as anaesthesia–resuscitation residents, and those who were instead in a “protected” situation, as in the case of psychiatry residents, did not show significant differences, neither with regard to anxiety (STAI I and II), nor for other indicators of psychological functioning and in general for the state of their health and well-being (the GHQ 12, SHS, SWLS and CISS). The only statistically significant differences are the values for resilience and depersonalisation, both of which are higher in anaesthetists. As far as anxiety is concerned, there is not a meaningful difference between the two specialities in percentage terms, but in the group comparison there seems to be a difference in awareness and probably the acceptance of it. In the anaesthetists’ group, the desire to minimise the presence and extent of anxiety prevailed, whereas in the psychiatrists’ comparison group, this did not occur. Yet, all trainees agreed that anxiety was not caused by the experience of the pandemic per se, not even for the anaesthetists who were on the front line and saw many people die in a short time, but was related to personal work aspects, in particular the assessment of their own competence. The pandemic, in the opinion of the trainees, complicated their learning and/or patient care pathways, and this exacerbated assessment anxieties, particularly for anaesthetists. In support of the interpretation of anxiety as not being directly related to the pandemic, we would like to stress the data relating to two experiences of particular emotional relevance, which involved anaesthesia–resuscitation residents during the pandemic: the experience of not being able to offer the most adequate care to everybody and the communication of worsening patient conditions. With regard to the former, we recall how, especially in the first wave, there was a dramatic choice over who to give respirators to, the number of which was less than the number of patients in need. Having experienced this, compared to those who had not been in that situation or had only rarely been in it, this did not differentiate the sample with respect to anxiety, but with respect to emotional exhaustion and depersonalisation. Similarly, the experience of reporting a deterioration in health conditions led to an increase in avoidance, social diversion and distraction, not anxiety. Confirming, on the other hand, the interpretation of anxiety as being more related to the assessment of competence is the finding that the perception of feeling or not feeling professionally qualified also differentiated the sample with respect to anxiety and well-being (GHQ 12, SHS and SWLS). Finally, we note that very few trainees sought psychological support. This finding is in line with that of another study, in which the percentage of professionals who worked with COVID-19 patients and asked for help was the same as our study [52].

In this work, a circumscribed and numerically limited population was studied, using various instruments. Although we are aware of the limitations of the work, some data nevertheless seem interesting to us, and may also be utilised for possible practical implications. The high percentage of anxiety in relation to self-assessment suggests that special attention should be paid to the learning experiences of trainees, possibly including moments of consolidation and comparison with respect to what has been acquired and experienced during the pandemic period. The increase in burnout indicators in relation to the unavailability of adequate medical facilities and care emphasises the role of well-functioning hospital departments. An efficient and effective healthcare system can also act as a buffer against burnout. This is an element to consider when rethinking the organisation of services after the pandemic. The increase in avoidance-oriented coping in highly emotional situations, such as communicating health deterioration, brings a relevant part of the physician’s work to the forefront. It is therefore considered important to supplement the learning of specific protocols for this communication (e.g., Buckman’s SPIKES) with the elaboration of the experiences involved in this activity. What has been highlighted here should be monitored over time, in order to verify, at a greater distance from the pandemic event and under different working conditions, which elements have remained constant, which have changed and which factors have determined their evolution. Research on larger samples and from different territories and cultures would be useful to assess how far the results are shared.

## 5. Conclusions

Two years after the dramatic beginning of the pandemic, partly positive and partly negative indicators are evident in this sample of trainees. In particular, their subjective happiness, satisfaction with their own life, a variety of defensive mechanisms and their resilience skills are good, but their widespread anxiety, psychopathological problems, depersonalisation, emotional exhaustion and their non-negligible use of emotion-oriented coping have also been encountered. Although the data are quite in line with the findings of other works, we have doubts that the data are in toto to be ascribed to the pandemic. There are in fact (except for depersonalisation and resilience) no significant differences between anaesthesia–resuscitation residents who practised with COVID-19 patients and psychiatrists who worked within more protected situations with a lesser workload, but above all, from the comparison with the residents, anxiety is attributed by all of them to the evaluative factors of their specialty pathway, not to the pandemic itself.

In fact, the most dramatic elements of the pandemic were identified as social isolation [53,54] and the fear of a SARS-CoV-2 infection [55,56], thus, in a broad sense, suffering, death and socio-economic problems [57,58]. Yet as far as the residents were concerned, the financial problems were not influential (indeed, the increased work was rewarded) and the presence on the ward and the support of the team contrasted the social isolation. A confrontation with suffering and death, even if not to the same degree, is somewhat implicit in the very choice of their speciality. It is plausible that the pandemic has had an impact, but from pre-pandemic mechanisms and arrangements.

In general, we feel that the trainees have found a certain balance, albeit one that can be improved. Instead, it seems to us that the recourse to avoidant coping mechanisms and the high presence of depersonalisation require careful monitoring. They may in fact be contingent responses, in which the pandemic and reduced work experience may have played a role, but since they are modalities that characterize early work experiences, they could constitute a lingering characterisation that would adversely affect the relationship with the patient. For this reason, rather than psychological support, the usefulness of which is perceived by only a small minority, the receipt of training that increases interpersonal skills and thereby offers support to operators, reducing the use of emotion-oriented coping, seems to us to be a significant investment in health, as operators and as users.

## Figures and Tables

**Table 1:** Percentages for the whole sample and in the separate samples of anaesthesia–resuscitation and psychiatry residents of the items of the ad hoc questionnaire.

	Total Sample	Anaesthesia–Resuscitation Trainees	Psychiatry Trainees
Women	43.3	42.9	44
Men	56.7	57.1	56
26–30 years old	76.7	74.6	81.5
31–39 years old	23.3	25.4	18.5
I live alone	30	33.3	22.2
I live with my partner	40	41.3	37
I live with my family of origin	20	20.6	22.2
I live with roommates	10	4.8	18.5
Direct experience of the disease (family, friends, colleagues)	77.8	77.8	77.8
Fear of infecting friends and relatives	68.9	74.6	55.6
Well supported by the team	52.2	52.4	51.9
Fairly supported by the team	40	42.9	33.3
More appreciation of life	32.3	31.7	33.3
Less desire to go out	32.4	31.8	33.3
I cultivate deeper relationships	24.4	22.2	29.6
I live a little more by the day	27.7	27	29.6
I feel open wounds	23.4	26.9	14.8
I bought a pet	8.9	7.9	11.1
I increased my sporting activity	24.4	27	18.5
Deterioration of physical health	24.4	27	18.5
Worsening psychological well-being	33.4	34.9	29.6
I increased my self-confidence	15.5	15.9	14.8
My self-confidence is stationary	47.8	46	51.9
Using sport to deal with emotionally involving work situations	31 (sometimes 34.5)	28.6 (sometimes 39.7)	33.3 (sometimes 18.5)
Using music to deal with emotionally involving work situations	32.9 (sometimes 38.8)	27 (sometimes 38.1)	40.7 (sometimes 33.3)
Using alcohol and the likes to deal with emotionally involving work situations	10.3 (sometimes 28.7)	12.7 (sometimes 30.2)	3.7 (sometimes 22.2)
Using spiritual practices to deal with emotionally involving work situations	9.3 (sometimes 23.3)	9.5 (sometimes 25.4)	7.4 (sometimes 14.8)
Sharing problems with others to deal with emotionally involving work situations	48.9 (sometimes 40)	49.2 (sometimes 38.1)	48.1 (sometimes 44.4)
Using professional help to deal with emotionally involving work situations	6.9 (sometimes 6.9)	4.8 (sometimes 7.9)	11.1 (sometimes 3.7)

**Table 2:** Mean standard deviation, percentiles and median, minimum and maximum values of the proposed tests.

	Mean (Standard Deviation)	Median	Minimum	Maximum
GHQ 12	17.37 (3.31)	17.00	10	26
Resilience	3.26 (0.72)	3.33	1.33	5
STAI I	44.29 (10.26)	42.50	20	67
STAI II	43.07 (9.15)	42.00	23	65
Emotional Exhaustion (EE)	19.61 (8.87)	19.50	2	41
Depersonalisation	12.12 (5.21)	12.00	2	22
Personal Accomplishment	27.08 (5.62)	27.00	14	40
SWLS	24.22 (5.71)	26.00	10	35
SHS	4.33 (1.28)	4.25	1	6.75
Task manoeuvre	50.42 (9.46)	50.50	25	75
Avoidance	51.97 (9.13)	51.50	31	69
Distraction	54.27 (9.28)	56.00	33	74
Social diversion	51.79 (9.32)	52.00	28	68
Emotion	50.46 (9.62)	51.00	29	75

GHQ—General Health Questionnaire; Resilience—Brief Resilience Scale; STAI I—State—Trait Anxiety Inventory I; STAI II—State -Trait Anxiety Inventory II; SWLS—Satisfaction with Life Scale; SHS—Subjective Happiness Scale; Task manoeuvre—Task-oriented.

**Table 3:** Cut-off point percentages in total sample, anaesthesia–resuscitation trainees, and psychiatry trainees.

Testing	Total Sample	Anaesthesia–Resuscitation Trainees	Psychiatry Trainees
GHQ12			
Low	12.2	14.3	7.4
High	87.8	85.7	92.6
Brief Resilience Scale			
Low	25.6	17.5	44.4
High	74.4	82.5	55.6
STAI I (state)			
Low	37.8	33.3	48.1
High	62.2	66.6	51.9
STAI II (trait)			
Low	41.1	42.9	37
High	58.9	57.1	63
Maslach Burnout Inventory			
Emotional exhaustion			
Low	30	23.8	44.4
Medium	40	49	15.6
High	30	27.2	37
Depersonalisation			
Low	1.1	0	3.7
Medium	32.2	23.8	51.9
High	66.7	76.2	44.4
Personal accomplishment			
Low	3.3	3	3.7
Medium	33.4	31.9	37
High	63.3	65.1	59.3
CISS			
Task-oriented			
Extremely below mean	1.1	1.6	0
Far below mean	6.7	6.3	7.4
Slightly below mean	41.1	47.7	25.9
Mean	1.1	1.6	0
Slightly above mean	35.6	31.8	44.5
Far above mean	13.3	11.1	18.5
Extremely above mean	1.1	0	3.7
Avoidance-oriented			
Extremely below mean	0	0	0
Far below mean	7.8	9.5	3.7
Slightly below mean	33.3	31.8	7.4
Mean	1.1	1.6	0
Slightly above mean	38.9	33.9	48.2
Far above mean	18.9	23.2	11.1
Extremely above mean	0	0	0
Social diversion			
Extremely below mean	2.2	3.2	0
Far below mean	4.4	4.8	3.7
Slightly below mean	35.5	36.5	26.3
Mean	3.3	3.2	3.7
Slightly above mean	31.1	27	40.8
Far above mean	23.3	25.4	18.5
Extremely above mean	0	0	0
Distraction			
Extremely below mean	0	0	0
Far below mean	7.8	9.5	3.7
Slightly below mean	22.2	22.2	22.2
Mean	2.2	1.6	3.7
Slightly above mean	42.2	36.5	55.6
Far above mean	23.4	27	11.1
Extremely above mean	2.2	3.2	0
Emotion-oriented			
Extremely below mean	1.1	0	0
Far below mean	12.2	14.3	3.7
Slightly below mean	31.1	31.7	25.9
Mean	4.4	4.8	3.7
Slightly above mean	33.3	31.7	37.1
Far above mean	17.8	15.9	22.2
Extremely above mean	1.1	1.6	0

GHQ—General Health Questionnaire; STAI I (state)—State-Trait Anxiety Inventory I; STAI II (trait)—State-Trait Anxiety Inventory II; CISS—Coping Inventory for Stressful Situations.

**Table 4:** Correlation between some indicators of health, well-being, burnout and coping strategies.

	Deterioration Phys. Health	Worsening Well-Being	SHS	SWLS	GHQ 12	Resilience
**Deterioration phys. health**		0.565 **	−0.293 **	−0.180	0.296 **	0.105
**Worsening well-being**	0.565 **		−0.386 **	−0.312 **	0.391 **	−0.218 *
**SHS**	−0.293 **	−0.386 **		659 **	−0.609 **	0.418 **
**SWLS**	−0.180	−0.312 **	659 **		−0.362 **	0.130
**GHQ 12**	0.296 **	0.391 **	−0.609 **	−0.362 **		−0.438 **
**Resilience**	−0.172	−0.218 *	0.418 **	0.130	−0.438 **	
**STAI I**	0.187	0.308 **	−0.649 **	−0.509 **	0.525 **	−0.439 **
**STAI II**	147	0.337 **			0.485 **	−0.506 **
**Emotional exhaustion**	0.371 **	0.392 **	−0.459 **	0.348 **	0.382 **	−0.286 **
**Depersonalisation**	0.301 **	0.216 *	−0.272 **	−0.349 **	−0.035	−0.081
**Personal accomplishment**	0.049	−0.118	0.246 *	0.281 *	−0.173	0.084
**Task-oriented**	0.030	0.001	0.378 **	0.426 **	−0.275 **	0.138
**Avoidance-oriented**	−0.012	−0.040	0.321 **	0.160	−0.258 *	0.072
**Social diversion**	0.002	−0.013	0.321 **	−0.210 *	−0.224 *	0.049
**Distraction**	−0.045	−0.046	0.140	0.044	−0.157	0.063
**Emotion-oriented**	0.181	0.230 *	−0.432 **	−0.374 **	0.352 **	−0.456 **

Spearman’s correlation. ** The correlation is significant at the 0.01 level (two-tailed). * The correlation is significant at the 0.05 level (two-tailed).

**Table 5:** Correlation between emotional exhaustion, depersonalisation, personal accomplishment and coping strategies.

	Emotional Exhaustion	Depersonalisation	Personal Accomplishment
**Avoidance-oriented**	−0.132	0.039	0.075
**Distraction**	−0.026	0.032	−0.013
**Social diversion**	−0.198	0.050	0.207
**Emotion-oriented**	0.431 **	0.277 **	−0.025
**Task-oriented**	0.105	−0.066	−0.312 **

Spearman’s correlation. ** The correlation is significant at the 0.01 level (two-tailed).

**Table 6:** Mean standard deviation and the median, minimum and maximum values of resilience (BRS) and depersonalisation in anaesthesia–resuscitation and psychiatry specializations.

	Resilience			Depersonalisation		
Specialisation	Mean (SD)	Median	Minimum Maximum	Mean (SD)	Median	Minimum Maximum
**Anaesthesia–Resuscitation**	3.39 (0.67)	3.33	2–5	13.10 (5.18)	13	4–22
**Psychiatry**	2.96 (0.74)	3	1.33–4.67	9.85 (4.61)	8	2–20
**Total**	3.26 (0.72)	3.33	1.33–5	12.12 (5.21)	12	2–22

**Table 7:** Mean standard deviation, median, minimum and maximum of SHS, GHQ 12, STAI I and STAI II values in the groups of anaesthetists differentiated with respect to the assessment of their professional competence.

Professional Qualification	N	Mean (SD)	Median	Min–Max
SHS The Subjective Happiness Scale				
high	6	4.29 (1.54)	3.87	2.25–6.25
medium	33	4.89 (1.00)	5.00	2.25–6.25
low	24	3.65 (1.47)	3.37	1–6.75
GHQ 12 General Health Questionnaire				
high	6	16.17 (2.78)	16.50	11–19
medium	33	16.39 (3.06)	16.00	11–25
low	24	18.62 (3.95)	17.50	10–26
STAI I State-Trait Anxiety Inventory I				
high	6	42.33 (15.46)	40.00	20–65
medium	33	42, 21 (8.35)	42.00	28–65
low	24	49.58 (9.69)	48	37–67
STAI II State-Trait Anxiety Inventory II				
high	6	42.33 (15.46)	40.00	20–65
medium	33	40.12 (7.95)	39.00	23–59
low	24	49.58 (9.69)	48.00	37–60

## Data Availability

The data are deposited in the university archive. Data will be available upon reasonable request.

## Supplementary Materials

The following supporting information can be downloaded at: https://www.mdpi.com/article/10.3390/ijerph192013136/s1. Supplementary file S1 Questionnaire.
