# Supplementary material for: Is This All COVID-19′s Fault? A Study on Trainees in One of the Most Affected Italian Cities
Source: Int J Environ Res Public Health. 2022 Oct 12;19(20):13136. doi: 10.3390/ijerph192013136 (PMC9603377; doi:10.3390/ijerph192013136)
Supplement: Supplementary file 1 [file ijerph-19-13136-s001.zip › ijerph-1879499-supplementary.pdf]

Questionnaire

Sex:
Age: ....
Who do you live with? Alone/ with roommates/ with a partner/ with family of origin
Have you had direct experiences (personal, family, friends) of illness? Yes No
If yes, please indicate for each one:
(who got sick?..
when? ....
Was there a need for hospitalisation? ....)
Has there been healing? ....
Have you ever been afraid of infecting family and friends?
Yes often Yes, sometimes No
Did you work in covid departments? Yes No
During your work did you experience not being able to offer everyone the most appropriate care?
Yes often Yes, sometimes No
Have you had to notify relatives of in-patients of the worsening health condition of a patient?
Yes, often Yes sometimes No
Did you generally feel qualified for the job you were asked to do? Yes Somewhat No
Did you feel supported by the work team? ... Yes Somewhat No
If you have experienced particularly intense emotional situations at work, how did you cope with them?

Sporting activity	Yes, often	Yes sometimes	No
Music	Yes, often	Yes sometimes	No
Alcohol	Yes, often	Yes sometimes	No

Spiritual' practices (prayer, meditation, yoga...)
Yes, often Yes sometimes No
Sharing with others (friends, colleagues, family)
Yes, often Yes sometimes No
Help from a professional Yes, often Yes Sometimes No
Other (specify)..
Please indicate to what extent you agree with each of the following statements, using the following scale:
1 = strongly disagree, 2 = disagree, 3 = neutral, 4 = agree, 5 = strongly agree
Following the pandemic....

I increased my sporting activity	1	2	3	4	5
I value life more	1	2	3	4	5
I feel less like going out	1	2	3	4	5
I have deeper relationships	1	2	3	4	5
I live more by the day	1	2	3	4	5
I feel open wounds in me	1	2	3	4	5
I bought an animal	1	2	3	4	5
I feel that my physical health has deteriorated (Psychological well-being)	1	2	3	4	5
I feel that my psychic well-being has deteriorated (Physical well-being)	1	2	3	4	5
I have greater self-confidence	1	2	3	4	5
